# Minimal effective weight-based dosing of ondansetron to reduce hypotension in cesarean section under spinal anesthesia: a randomized controlled superiority trial

**DOI:** 10.1186/s12871-018-0568-7

**Published:** 2018-08-15

**Authors:** Maliwan Oofuvong, Thitikan Kunapaisal, Orarat Karnjanawanichkul, Nussara Dilokrattanaphijit, Jaranya Leeratiwong

**Affiliations:** Department of Anesthesiology, Faculty of Medicine, Prince of Songkla University, 15 Kanjanavanich Road, Songkhla, 90112 Thailand

**Keywords:** Weight-based dosing, Ondansetron, Hypotension, Spinal anesthesia, Cesarean section

## Abstract

**Background:**

The weight-based dosing of ondansetron to reduce hypotension has never been investigated. The aim of this study is to determine the optimal dose of ondansetron required based on the patient’s weight to reduce hypotension following spinal anesthesia for cesarean section.

**Methods:**

In this prospective, triple-blinded, parallel group, randomized controlled trial, a total of 228 pregnant women were randomized to receive either normal saline (group NS) or ondansetron 0.05 mg/kg (group O1) or ondansetron 0.1 mg/kg (group O2) intravenously 5 min before induction of spinal anesthesia. The incidence of hypotension, mean arterial pressure, heart rate, vasopressor requirements, and blood loss between the three groups were compared. Maternal and neonatal complications were also assessed. Changes in blood pressure and heart rate were compared using the generalized estimating equations method.

**Results:**

Thirteen patients were excluded from the analysis because of no intervention (*n* = 12) and protocol violation (*n* = 1). Two hundred and fifteen patients remained for the intention-to-treat analysis. The incidence of hypotension in groups NS (*n* = 72), O1 (*n* = 71), and O2 (*n* = 72) were 81.9%, 84.5%, and 73.6%, respectively (*P* = 0.23). The episodes of hypotension before delivery (first 14 min after spinal anesthesia) were significantly higher in group O1 compared to NS (5 vs 2, *P* = 0.02). The overall heart rates throughout the operations were not different among the three groups. The ephedrine requirements and amount of blood loss were also similar among the three groups. The metoclopramide requirement was significantly lower in group O2 compared to group NS (2.8% vs 16.7%, *P* = 0.01). There were no serious adverse events in terms of maternal or neonatal complications.

**Conclusions:**

Ondansetron 0.05 mg/kg or 0.1 mg/kg administered before spinal anesthesia did not reduce the incidence of hypotension in this study.

**Trial registration:**

Thai Clinical Trials Registry, TCTR 20160323001, 22 March 2016.

## Background

Spinal anesthesia is now the technique of choice for women undergoing cesarean section. However, hypotension is a common problem after surgery with reported incidences varying from 53 to 85% worldwide [1, 2]. The Bezold–Jarisch reflex, which occurs after spinal anesthesia, induces vasodilatation and decreases venous return which results in bradycardia and hypotension [3]. A systematic review reported that neither intravenous fluid preload nor vasoconstriction given before spinal anesthesia is effective in preventing hypotension [4]. One factor known to influence the Bezold–Jarisch reflex is serotonin. Ondansetron is a serotonin receptor blocker. It decreases serotonin induced by the Bezold–Jarisch reflex by suppressing venodilatation [5]. Two studies found that ondansetron given at a dose of 8 mg before spinal anesthesia could reduce hypotension by 86% in general surgery [6] and by 33% in cesarean section [7]. However, another study showed that the same dose of ondansetron could not significantly reduce hypotension in pregnant women [8]. Fattahi et al. [9] showed that the mean arterial pressure after spinal anesthesia in patients who had a cesarean section was significantly higher in women given ondansetron 0.15 mg/kg compared to the control group. Sahoo et al. [10] reported the lower dose of ondansetron (4 mg) compared to control group could reduce hypotension in parturients in cesarean section. In terms of safety, Pasternak et al. [11] and Einarson et al. [12] showed that ondansetron administered in over 600,000 pregnant women had no significant effect on the newborn.

All of these studies used a fixed dose of ondansetron. To our knowledge, the optimal dose based on the patient’s weight has never been investigated. Using a weight-based dose of ondansetron might maximize its effect and prevent suboptimal dose or overdose in some parturients since they have a larger volume of distribution compared to normal patients. Hence, this study aims to determine the minimal weight-based dose of ondansetron required to reduce hypotension after spinal anesthesia in women undergoing cesarean section.

## Methods

This was a prospective triple-blinded parallel randomized controlled superiority trial. The study was approved by the Institutional Ethics Committee of The Faculty of Medicine, Prince of Songkla University, Songkhla, Thailand on March 3, 2016 (EC 58382081), Clinicaltrials.in.th number TCTR 20160323001 on March 22, 2016.

### Participants

A written informed consent was obtained from all participants. The eligible participants were singleton pregnant women at a gestational age of 37–42 weeks who underwent elective cesarean section under spinal anesthesia and had no abnormal signs on electrocardiography. The study was carried out between May 2016 and February 2017 at Songklanagarind Hospital which is an 853–bed tertiary care hospital in southern Thailand. The exclusion criteria included the inability to communicate, history of hypertension, history of being allergic to ondansetron, congenital abnormality or acquired heart disease, coagulopathy, morbid obesity (body mass index > 35 kg/m^2^), abnormal pregnancy with intrauterine growth retardation, congenital anomalies, poly- or oligo-hydramnios, and placenta previa. These criteria were meant to exclude patients who had a high risk of hemodynamic instability.

### Standard operating procedures

After obtaining written informed consent from the participants by TK, baseline electrocardiography was performed and the women were randomized with a 1:1:1 allocation ratio to receive intravenously either normal saline (group NS) or ondansetron 0.05 mg/kg (group O1) or ondansetron 0.1 mg/kg (group O2) (maximal dose of 8 mg). Block randomization was performed in blocks of three by computer generated tables by a hospital research assistant who was not involved in the trial. A 4 mL clear solution was prepared for all study participants in sequential numbers by a nurse anesthetist in the recovery room who was not involved in the operation. The vial containing the solution was put into a concealed envelope for the nurse anesthetist who was in charge in the operating room. The syringes had no identifying markers indicating group allocation. The patient, anesthesiologist in charge, and the investigators were blinded to the group allocation. The intention-to-treat protocol was applied in this study. Premedication by ranitidine or metoclopramide or both could be given according to the decision of the anesthesiologist in charge.

In the operating room, 1000 mL of isotonic crystalloid was given to every patient. Philips IntelliVue MX700 was used to monitor non-invasive blood pressure, pulse oximetry, and electrocardiography during the period of anesthesia. Before spinal anesthesia was performed, the 4 mL clear solution containing the allocated treatment was administered intravenously five min before spinal anesthesia. Spinal anesthesia was performed at level L2–L3 or L3–L4 in the vertebral space with 2.0 mL of 0.5% hyperbaric bupivacaine and 0.2 mg of intrathecal morphine at a rate of 0.2 mL/sec by an experienced resident anesthetist. After spinal anesthesia was performed, systolic blood pressure (SBP), diastolic blood pressure (DBP), and mean arterial blood pressure (MAP) were recorded every one min for 15 min and then every five minutes, while the heart rates were recorded every five minutes until the end of the operation by a nurse anesthetist who was masked to the group allocation. Left uterine displacement of 15 to 20 degrees was performed after spinal anesthesia by placing a wedge under the right hip until the baby was delivered. After the baby was delivered, oxytocin (20 units per 1000 mL of isotonic crystalloid) was routinely given while methylergometrine would be given only upon request of the gynecologist surgeon. The doses of oxytocin and methylergometrine were measured.

### Outcomes of the study

The primary outcomes of the study were the incidence of hypotension and the change in MAP. Hypotension was defined as a decrease in MAP > 30% from baseline. If hypotension occurred, 6 mg of ephedrine or 5 mcg of norepinephrine was administered intravenously according to the discretion of the anesthesiologist in charge.

The secondary outcomes were maternal complications during the intraoperative period and in the post-anesthetic care unit and neonatal complications 24 h postoperatively. If itching occurred, 10 mg of chlorpheniramine was given intravenously. Metoclopramide (10 mg) was given intravenously for treatment of nausea and vomiting during the intraoperative period. Any side effects from ondansetron such as burning and QT prolongation were evaluated. The QT prolongation was determined by a QT interval more than half of the preceding RR interval in 3-lead electrocardiogram monitoring intraoperatively and at post-anesthetic care unit. The well-being of the newborn baby was evaluated as well as any adverse events 24 h postoperatively.

### Sample size determination

The sample size calculation was based on a difference in the incidence of hypotension between the control group (0.85) and the ondansetron group (8 mg) from a previous study which reported the incidence as 0.6 [7]. For a power of 90% to detect this difference and a type I error rate of 0.05, 73 patients per group were required under the assumption that 10% of the study participants would withdraw from the study.

### Statistical analysis

Continuous variables are presented as median and interquartile range for non-normally distributed data or mean and standard deviation for normally distributed data. Categorical variables are presented as frequency and percentage. Continuous variables were compared using two-way analysis of variance and the Kruskal–Wallis test for normally and non-normally distributed data, respectively. Categorical variables were compared using Pearson’s Chi-square test or Fisher’s exact test for normally and non-normally distributed data, respectively. Post-hoc analysis was performed for multiple comparisons between groups when the overall differences were significant. Changes in SBP, DBP, MAP, and heart rate were compared using the generalized estimating equations method. A *P* value < 0.05 was considered statistically significant. Group allocation was also blinded to the statistician who analyzed the data.

## Results

**Fig. 1:**
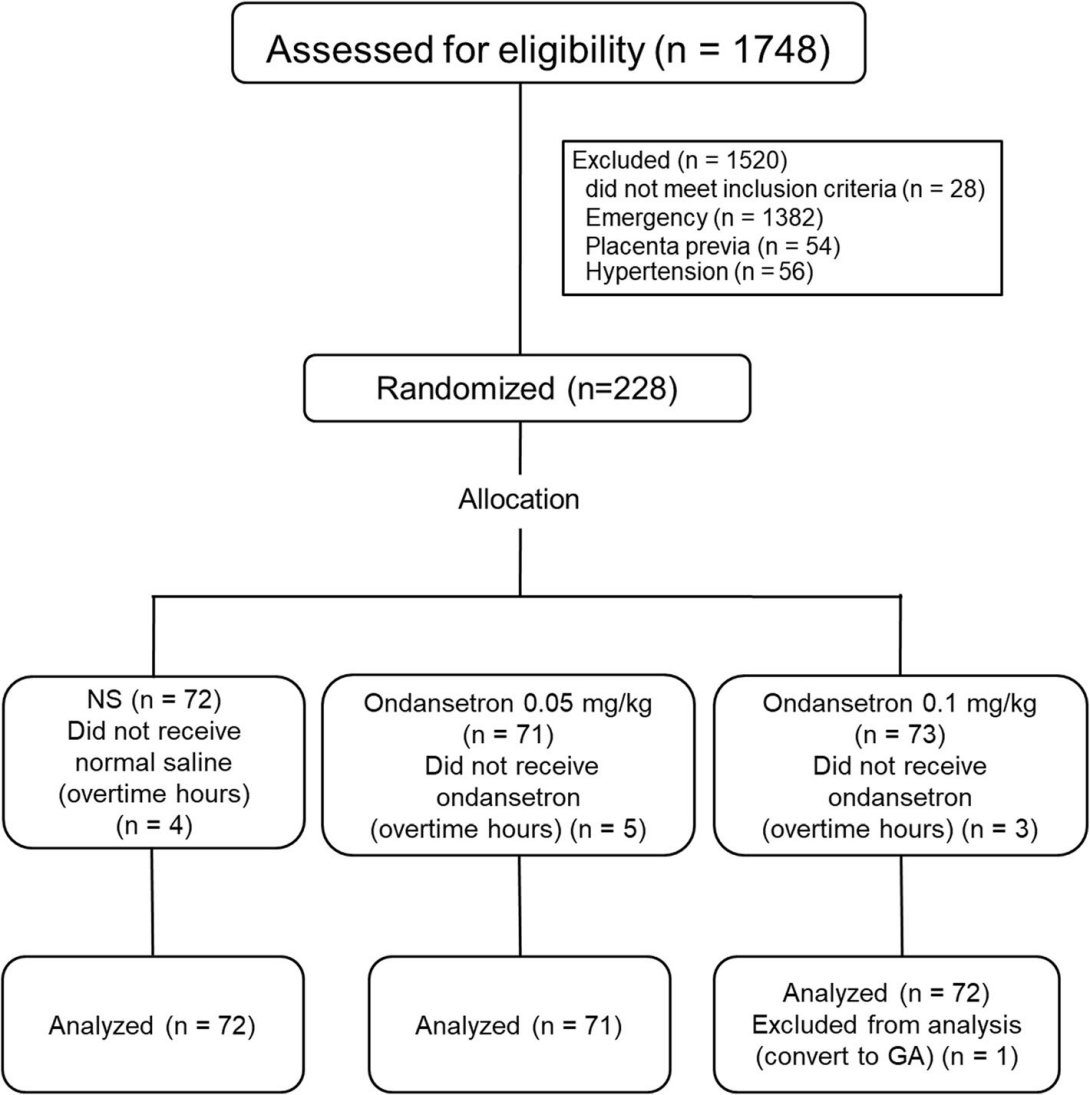
Consort flow diagram. *GA* general anesthesia

**Fig. 2:**
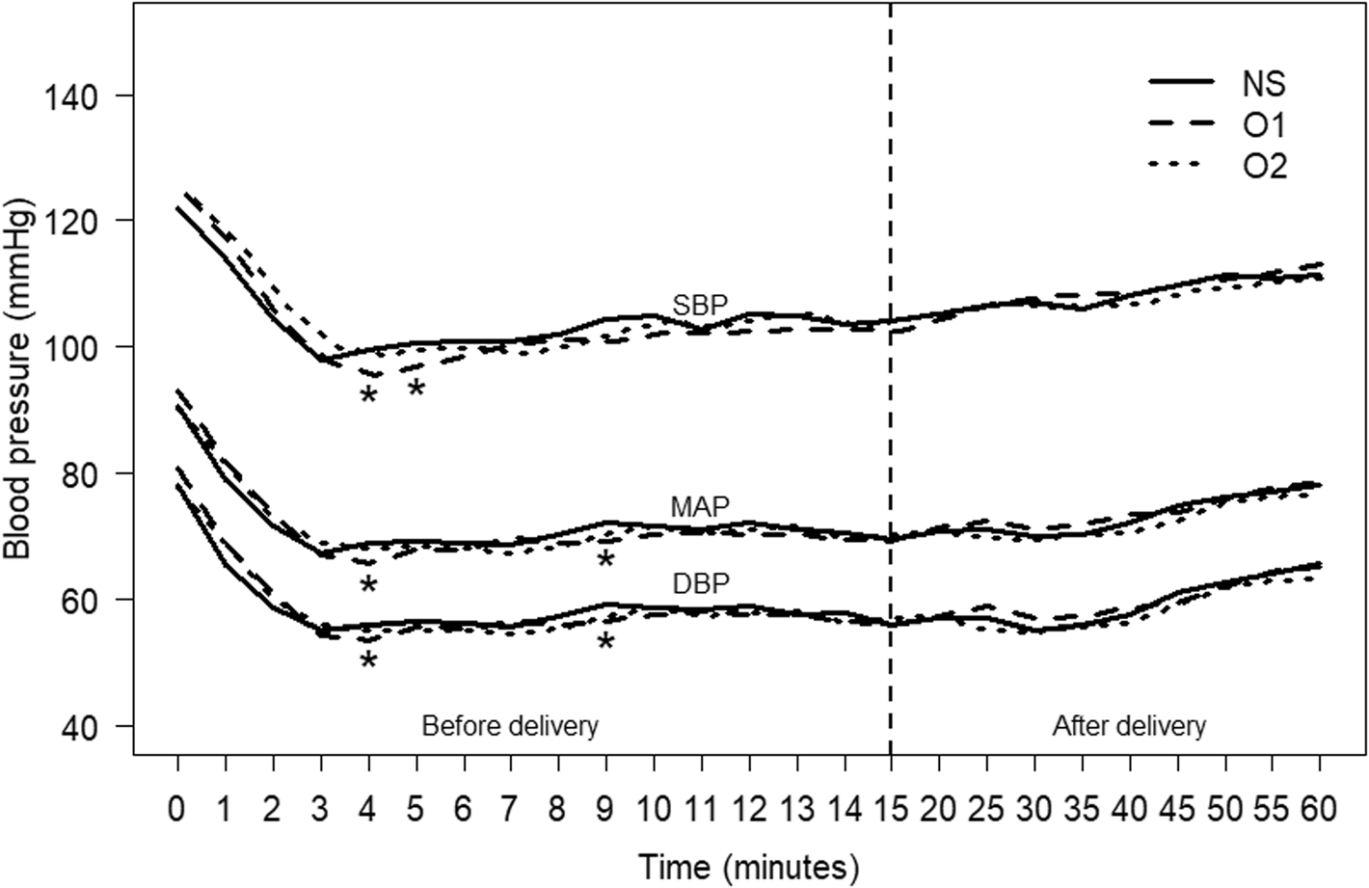
Changes in maternal systolic blood pressure (SBP), diastolic blood pressure (DBP) and mean arterial pressure (MAP) for the three groups. *NS* normal saline, *O1* ondansetron 0.05 mg/kg, *O2* ondansetron 0.1 mg/kg. Spinal anesthesia was performed between Time 0–1, Time 0 = baseline blood pressure, Time 1–60 = 1 to 60 min after spinal anesthesia, *P < 0.05 compared group O1 to NS at a certain time compared to Time 0

**Fig. 3:**
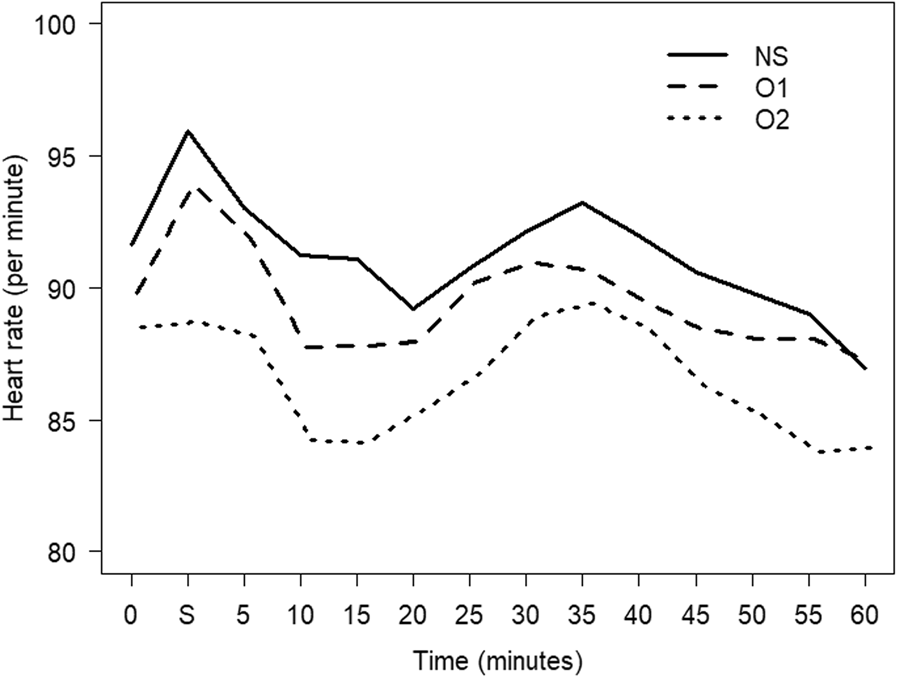
Changes in heart rate for the three groups. *NS* normal saline, *O1* ondansetron 0.05 mg/kg, *O2* ondansetron 0.1 mg/kg. Time 0 = baseline heart rate, Time S = spinal anesthesia and 5 min after received intervention, Time 5–60 = 5 to 60 min after spinal anesthesia

Two hundred and twenty-eight women were enrolled in the study with details shown in the consort diagram in Fig. 1. Data collection was completed in 215 patients for the intention-to-treat analysis. Patient characteristics and anesthetic data are presented in Table 1. No differences among the groups were observed in demographic data and anesthetic data. The overall incidence of maternal hypotension was 80%. There were no differences in the incidence of hypotension between the three groups either before or after delivery except episodes of hypotension before delivery were significantly higher in group O1 compared to NS (5 vs 2, *P* = 0.02) (Table 2). The arterial pressures (SBP, MAP, and DBP) are shown in Fig. 2. MAP levels at 4 and 9 min after spinal anesthesia were significantly lower in group O1 compared to the NS group (*P* = 0.024 and *P* = 0.034, respectively). The overall heart rates throughout the operations were not different among the three groups (Fig. 3). Ephedrine requirements were similar among the three groups: group NS (6 mg), group O1 (12 mg), and group O2 (6 mg) (*P* = 0.21). There were also no differences in blood loss among the three groups: group NS (447.9 ± 201.3 mL), group O1 (490.8 ± 241.2 mL), and group O2 (440.3 ± 198.2 mL) (*P* = 0.32). Table 3 shows the surgical and anesthetic parameters for the three groups. The metoclopramide requirement in the post-hoc analysis in group O2 was significantly lower than in group NS (*P* = 0.01). Table 4 compares the incidence of maternal and newborn adverse events between the three groups. No significant differences were found although tachypnea was slightly higher among newborns of women who received low dose ondansetron (7.0%, *n* = 5) compared to the control group (2.8%, *n* = 2) (*P* = 0.28). There were no reports of burning or QT prolongation in mothers and no reports of bradycardia or hypothermia in newborns in any group.

**Table 1:** Comparison of demographic and anesthesia related data between the three groups

Parameter	Normal saline (*n* = 72)	Ondansetron 0.05 mg/kg (*n* = 71)	Ondansetron 0.1 mg/kg (*n* = 72)	*P-*value
Age (years)^a^	31.8 (4.5)	32.2 (5.5)	32.7 (4.8)	0.55
Weight (kg)^a^	67.5 (9.6)	68.7 (9.1)	69.6 (8.9)	0.39
Height (cm)^a^	157.2 (5.5)	156.9 (5.7)	157.9 (5.2)	0.48
Body mass index (kg/m^2^)^a^	27.3 (3.5)	27.9 (3.0)	27.9 (3.2)	0.47
Type of operation				
CS only	49 (68.1)	49 (69)	46 (63.9)	0.79
CS with tubal resection	23 (31.9)	22 (31)	26 (36.1)	
Site of spinal anesthesia				
L2–3	2 (2.8)	0 (0)	0 (0)	0.33
L3–4	70 (97.2)	71 (100)	72 (100)	
Number of blocks				
1	70 (97.2)	70 (98.6)	69 (95.8)	0.87
2	2 (2.8)	1 (1.4)	3 (4.2)	
Anesthesia level^b^	T7 (T6–T8)	T6 (T6–T8)	T6 (T6–T8)	0.33
Analgesia level^b^	T4 (T3–T4)	T3 (T3–T4)	T4 (T3–T4)	0.43
Premedication with metoclopramide	25 (34.7)	21 (29.6)	21 (29.2)	0.73
Premedication with ranitidine	25 (34.7)	21 (29.6)	21 (29.2)	0.73

Data are presented as frequency (%) unless stated otherwise

^a^mean (SD)

^b^median (IQR)

*CS* cesarean section

**Table 2:** Comparison of incidence and episodes of hypotension between the three groups

Parameter	Normal saline (*n* = 72)	Ondansetron 0.05 mg/kg (*n* = 71)	Ondansetron 0.1 mg/kg (*n* = 72)	*P-*value
Total incidence,				
*n* (%)	59 (81.9)	60 (84.5)	53 (73.6)	0.23
(95% CI)	(73.1–90.8)	(76.1–92.9)	(63.4–83.8)	
Episodes, median (IQR)	2 (0–4.0)	3 (0.5–5.0)	1 (0–3.2)	0.07
Before delivery				
Incidence,				
*n* (%)	57 (79.2)	59 (83.1)	50 (69.4)	0.13
(95% CI)	(69.8–88.5)	(74.4–91.8)	(58.8–80.1)	
Episodes, median (IQR)	2 (1–6.0)^a^	5 (1.5–8.0)^b^	2 (0–5.0)^ac^	0.015
After delivery				
Total incidence,				
*n* (%)	37 (52.1)	41 (57.7)	39 (54.9)	0.80
(95% CI)	(40.5–63.7)	(46.3–69.2)	(43.4–66.5)	
Episodes, median (IQR)	0 (0–2.0)	1 (0–3.0)	1 (0–1.5)	0.64

*IQR* interquartile range, *CI* confidence interval

^ac^Groups sharing the same superscript were not significantly different, ^b^Group was significantly different compared to the others

**Table 3:** Comparison of surgical and anesthetic parameters between the three groups

Parameter	Normal saline (*n* = 72)	Ondansetron 0.05 mg/kg (*n* = 71)	Ondansetron 0.1 mg/kg (*n* = 72)	*P*-value
Duration of surgery (min)	56.6 (18.1)	58.9 (22.3)	55.2 (16.5)	0.52
Ephedrine (mg), median (IQR)	6 (0–18)	12 (0–18)	6 (0–18)	0.21
Norepinephrine (mcg), median (IQR)	0 (0–10)	0 (0–10)	0 (0–10)	0.47
Oxytocin (unit)	22.7 (8.5)	21.3 (9.1)	22.4 (9.7)	0.62
Methylergometrine (mg), median (IQR)	0 (0–0.2)	0 (0–0.2)	0 (0–0.2)	0.77
Blood loss (mL)	447.9 (201.3)	490.8 (241.2)	440.3 (198.2)	0.32
Crystalloid (mL)	1934.7 (303.3)	1884.5 (346.2)	1891.7 (284.8)	0.58
Packed red cells (mL)	0 (0)	2.8 (23.9)	0 (0)	0.36
Metoclopramide, n (%)	12 (16.7)^a^	6 (8.5)^ab^	2 (2.8)^b^	0.02

Data are presented as mean (SD) unless stated otherwise

^ab^Groups sharing the same superscript were not significantly different

*IQR* interquartile range

**Table 4:** Comparison of maternal and newborn adverse events between the three groups

Adverse event	Normal saline (*n* = 72)	Ondansetron 0.05 mg/kg (*n* = 71)	Ondansetron 0.1 mg/kg (*n* = 72)	*P*-value
Mother				
Urticaria	1 (1.4)	0 (0)	2 (2.8)	0.78
Arrhythmia	2 (2.8)	0 (0)	1 (1.4)	0.78
Vomiting	10 (13.9)	10 (14.1)	3 (4.2)	0.09
Itching	10 (13.9)	4 (5.6)	7 (9.7)	0.25
Newborn				
Apgar 1, median (IQR)	9 (8–9)	9 (8–9)	9 (8–9)	0.94
Apgar 5, median (IQR)	9 (9–9)	9 (9–9)	9 (9–9)	0.14
Hypotension	0 (0)	1 (1.4)	0 (0)	0.33
Tachypnea	2 (2.8)	5 (7.0)	0 (0)	0.04*

Data are presented as frequency (%) unless stated otherwise

*no differences for multiple comparisons between groups (*P* > 0.05)

## Discussion

In this study, we found that a weight-based dose of prophylactic ondansetron did not influence the incidence of maternal hypotension. The results of this study were consistent with two other studies [8, 13] but were in contrast with others [6, 7, 9, 10]. Two recent systematic reviews [14, 15] showed ondansetron reduced the incidence of hypotension (relative risk ratio [RR] = 0.63, 95% confidence interval [CI]: 0.45–0.88 and RR = 0.67, 95% CI: 0.54–0.83, respectively) and bradycardia (RR = 0.31, 95% CI: 0.19–0.50 and RR = 0.49, 95% CI: 0.28–0.87, respectively) after spinal anesthesia in cesarean section even though a low quality of evidence was reported [15]. However, the minimal effective weight-based dosing of ondansetron in parturients was not determined [9, 10, 14, 15].

In our study, ondansetron had little effect on hemodynamic stability after subarachnoid block in parturients. Factors that impact the level of anesthesia and analgesia given to the patient might also affect the patient’s arterial pressure as well as the way to measure arterial pressure. There are some possible reasons for the difference in the incidence of hypotension compared to other reports. First, we did not monitor invasive arterial blood pressure or cardiac output. Real time arterial pressure monitoring may be better at detecting actual variations of blood pressure than noninvasive blood pressure monitoring although we measured blood pressure every minute during the first 15 min after spinal anesthesia. A noninvasive blood pressure measurement might not be sufficiently sensitive to notice hemodynamic changes to promptly treat hypotension in parturients, especially in preeclamptic patients [16]. Langesaeter et al. [17] and Rosseland et al. [18] showed that continuous invasive arterial pressure monitoring during cesarean section under subarachnoid block provided hemodynamic stability in parturients, especially when vasopressor was given. Second, vasodilatation from subarachnoid block might have a stronger influence on blood pressure than the Bezold–Jarisch reflex in our study. From the results, 0.05 mg of ondansetron caused more episodes of hypotension before delivery compared to the control group (*P* = 0.02), especially at 4 and 9 min after spinal anesthesia (Fig. 2) which led to failure to prevent the Bezold–Jarisch reflex. Therefore, ondansetron may have little effect on preventing hypotension in these cases since we administered a vasopressor to treat vasodilatation immediately after hypotension occurred causing no differences in the incidence of hypotension among the three groups. We recorded blood pressure after a subarachnoid block was performed every minute for 15 min. If hypotension occurred, ephedrine or norepinephrine was administered instantly until the MAP reached the lower limit of baseline level. The peak onset of ephedrine or norepinephrine is approximately 1–2 min [19] and repeated doses were given as needed. Third, the weight-based dosing of 0.1 mg/kg of ondansetron might not be adequate to maximize the effect to prevent the Bezold–Jarisch reflex. A high dose of ondansetron (12 mg) or 0.15 mg/kg of ondansetron was reported to increase MAP in patients who had a cesarean section under spinal anesthesia compared to the control group [9, 20]. In our study, the mean dose of 3 to 7.9 mg of ondansetron was given which might not be adequate to prevent the Bezold–Jarisch reflex even though some studies found a lower dose of ondansetron (4 or 6 mg) could significantly prevent hypotension in the same setting [10, 20]. However, we were concerned that the dose of 0.15 mg/kg of ondansetron might be harmful to the fetus in terms of possible umbilical arterial vasoconstriction due to ondansetron acting on the 5-HT1B receptor [21]. Fourth, our analysis was based on an intention-to-treat principle which included repeat administration of subarachnoid blocks following failure of the first spinal anesthesia. A repeat dose of subarachnoid block can affect the level of anesthesia and analgesia, although there were no significant differences in the number of blocks given between the three groups (*P* = 0.87).

The definition of hypotension used in this study was a decrease in the MAP by 30% from the baseline level which was quite similar to many studies [7, 8, 22, 23]. MAP was used instead of SBP to define hypotension because MAP provides a physiologically more appropriate measurement of hypotension than SBP [24]. The overall incidence of hypotension among women undergoing cesarean section following spinal anesthesia in our study (80%) was lower than reported in a previous study done in Songklanagarind Hospital (85%) [2]. One-third of each intervention group received metoclopramide as premedication. Even though a case report showed 10 mg of metoclopramide could cause severe hypotension [25], the baseline blood pressure before spinal anesthesia was not different among the three groups (Fig. 2).

Ondansetron did not cause any adverse events in our study. The parturients experienced neither QT abnormalities nor burning sensation during the injection and the newborns did not experience bradycardia or hypothermia 24 h postoperatively. The incidence of urticaria or arrhythmia was not higher in the two ondansetron groups. Although the incidence of vomiting was lower in the high dose ondansetron (0.1 mg/kg) group, the incidence was not statistically different (*P* = 0.09). However, postoperative metoclopramide requirement was significantly lower in the high dose ondansetron compared to the normal saline group (*P* = 0.01). Therefore, the effect of ondansetron to prevent or treat nausea and vomiting was achieved as expected. There were no significant differences in terms of hypotension or tachypnea in newborns between the three groups. The incidence of tachypnea was slightly higher among the newborns of women who received the low dose of ondansetron compared to the control group (7% vs 2.8%). Tachypnea resolved within 2–3 h after oxygen therapy was administered. Although ondansetron can transfer across the placenta to newborn babies [26], adverse events related to ondansetron in newborns have not been reported.

### Strengths and limitations

The strengths of this study were reduced selection bias due to the randomized treatment allocation and the triple-blind design (patients, investigators, and statistician). We used the intention-to-treat principle in the analysis which helped decrease the bias and ensure a balance of patients between the three groups. There are two weaknesses of this study. First, several resident anesthetists performed the spinal block which might have affected the level of anesthesia and incidence of hypotension in the study participants. However, these residents had a high level of experience (> 20 spinal blocks) and a fixed rate of subarachnoid injection (0.2 mL/s) was used in all participants. Second, we did not monitor invasive arterial blood pressure because invasive monitoring is not routinely used in healthy parturients in our setting and it is also costly. However, the accuracy of the study is high and reproducibility of the results for a similar setting (a tertiary care hospital) should be achievable even though the study was conducted in a single hospital.

## Conclusions

Ondansetron administered at a dose of 0.05 mg/kg or 0.1 mg/kg before spinal anesthesia was not effective in reducing the incidence of hypotension in pregnant women undergoing cesarean section. Higher weight-based dosing of ondansetron should be examined keeping the safety issues in mind.

## Abbreviations

CI: Confidence interval
DBP: Diastolic blood pressure
MAP: Mean arterial blood pressure
RR: Relative risk ratio
SBP: Systolic blood pressure
